# Shape shifting by amphibious plants in dynamic hydrological niches

**DOI:** 10.1111/nph.16347

**Published:** 2019-12-31

**Authors:** Hans van Veen, Rashmi Sasidharan

**Affiliations:** ^1^ Plant Ecophysiology Institute of Environmental Biology Utrecht University Padualaan 8 3584 CH Utrecht the Netherlands

**Keywords:** amphibious, bicarbonate, carbon‐concentrating mechanisms, drought, flooding, leaf development, plasticity

## Abstract

Amphibious plants thrive in areas with fluctuating water levels, partly as a result of their capacity to make specialized leaves when submerged or emerged. The tailor‐made leaves improve gas exchange underwater or prevent aerial desiccation. Aquatic leaves are thin with narrow or dissected forms, thin cuticles and fewer stomata. These traits can combine with carbon‐concentrating mechanisms and various inorganic carbon utilization strategies. Signalling networks underlying this plasticity include conserved players like abscisic acid and ethylene, but closer inspection reveals greater variation in regulatory behaviours. Moreover, it seems that amphibious leaf development overrides and reverses conserved signalling pathways of their terrestrial counterparts. The diversity of physiology and signalling makes plant amphibians particularly attractive for gaining insights into the evolution of signalling and crop improvement.

## Abstract 2

See also the Editorial on this article by Sasidharan et al., **229**: 5–7.

**Table:** 

**Contents**		
	Summary	79
I.	Introduction	79
II.	Leaf morphological transitions	80
III.	Underwater photosynthesis	82
IV.	Conclusions and future challenges	83
	Acknowledgements	83
	References	83

## I. Introduction

Hydrological gradients are a strong determinant of plant species distribution, and species occupying the riparian side of these gradients experience fluctuating water levels and high flooding risks (Silvertown *et al*., 2015; Sarneel *et al.*, 2019). For plants that are used to terrestrial life, inundation has dramatic consequences (Loreti *et al.*, 2016). In the aquatic environment, gas diffusion is *c.* 10 000 times slower, which has grave consequences for oxygen (O_2_) and carbon dioxide (CO_2_) availability (Nobel 2009). Combined with potential reductions in light availability underwater, photosynthesis will be severely hampered. The resulting energy and carbon crisis is perhaps the greatest challenge for flooded plants. In illuminated conditions, the reduction in photosynthesis also generates oxidative stress as a result of an imbalance between light‐harvesting and diffusion‐limited carbon fixation (Horiguchi *et al.*, 2019). For plants that typically inhabit the aquatic niche, the sudden aerial exposure is also not without risk. The lack of a thick cuticle makes their leaves prone to desiccation and the sudden exposure to light and high amounts of O_2_ lead to excessive reactive oxygen species formation (Yeung *et al.*, 2018).

Amphibious plants can successfully occupy the terrestrial–aquatic environmental interface. They often propagate via tubers and rhizomes, and/or time their life cycle to coincide with periods of favourable water levels (Sosnova *et al.*, 2010). During shallow flooding, elongation of shoot organs can facilitate re‐establishment of aerial contact and is typically combined with aerenchyma formation to improve internal aeration (Pierik *et al.*, 2008; Herzog & Pedersen 2014).

Despite possessing leaves with a slightly higher specific leaf area (Box 1), species from the water’s edge do not have better underwater photosynthesis than those from higher elevation levels (Winkel *et al.*, 2016). However, many species living in this transition zone do have the capacity to form new leaves adapted to either the new aquatic or aerial conditions. This drastic alteration of leaf form in response to environmental changes is termed heterophylly.

Box 1 **Glossary of specialist terms used in this article**

**Specific leaf area (SLA):** The amount of leaf area per unit leaf mass (m^2^ kg^−1^). It is considered a major factor associated with plant growth variation. As high SLA is associated with thin leaves, it is considered an important trait for underwater photosynthesis. In this case, diffusion distance is lower. Thus high‐SLA leaves typically have better gas exchange.

**Boundary layer:** A stationary fluid layer immediately covering the surface of submersed objects (e.g. flooded plant organs). No bulk flow of the liquid is observed in this layer, so movement of all compounds is driven solely by diffusion. This layer therefore severely impairs underwater gas exchange. The thickness of the diffusive boundary layer is dependent on the flow rate of the water and the surface topography of the submerged object. In the context of submerged plants, thicker boundary layers are expected in still or slow‐moving water and especially on large leaves.

**Kranz anatomy:** Distinctive leaf anatomy associated with C_4_ photosynthesis. Typical ‘Kranz’ (German for ‘wreath’) structure includes an outer ring of mesophyll cells and an inner ring of bundle sheath cells surrounding the vascular tissues.

**Heterophylly:** The formation of extremely different leaf forms on a single plant. These leaf forms are created early in the development of a leaf. The extreme variation can be induced by a variety of factors such as age, temperature and humidity. In this paper, we refer to alterations caused by transition between flooded and aerial conditions.

**True aquatics:** Plant species that are adapted to submerged conditions and can only survive underwater.

Aquatic leaves, compared with those formed aerially, usually show a higher amount of dissection or they retain the simple leaf shape with a more narrow, elongated form. Additional changes in aquatic leaves include a minimal or even absent cuticle, and fewer or absent stomata. Besides the remarkable display of leaf plasticity, some amphibious plants utilize carbon‐concentrating mechanisms (CCMs) and/or bicarbonate (HCO_3_^–^) uptake systems to improve underwater photosynthesis and facilitate the amphibious dual life. A multitude of internal and external signals are used to sense air–water transitions and trigger these dramatic changes. Here we highlight the current understanding of shoot plasticity and photosynthesis physiology, and their adaptive significance for an amphibious lifestyle. We also call for increased leveraging of wild species for broadening our knowledge on mechanisms of plasticity in variable environments.

## II. Leaf morphological transitions

**Figure 1:**
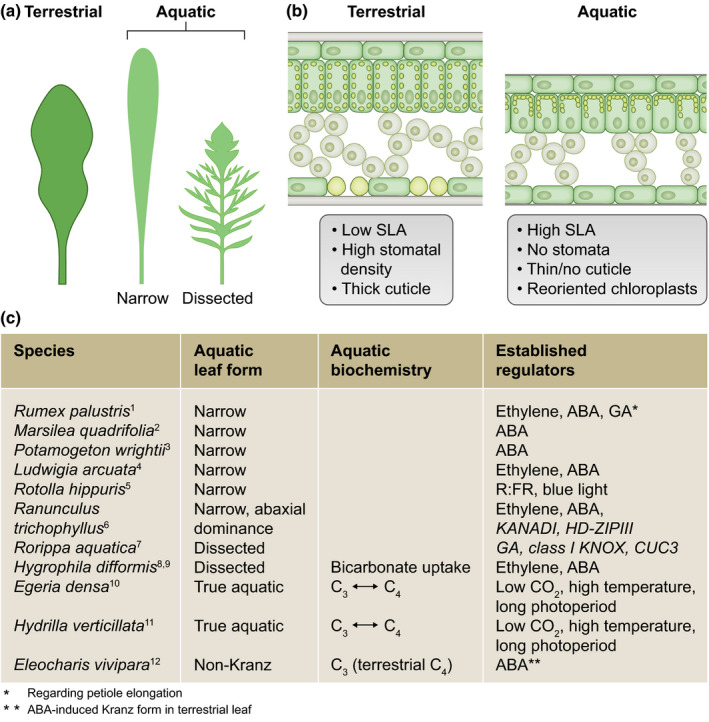
Shoot adaptive plasticity in amphibious plants. (a) General illustration of distinct terrestrial and aquatic leaf forms of amphibious species. Aquatic leaves tend either to be slightly elongated and narrow or to have a strongly dissected form. (b) Cross‐section of a typical terrestrial and aquatic leaf. Terrestrial leaves are thicker, with a thick cuticle and predominantly abaxial localized stomata. Aquatic leaves have features to enhance underwater gas exchange capacity. They tend to be much thinner (high specific leaf area, SLA) (Box 1), with a reduced or absent cuticle, larger air spaces, little or no stomata and chloroplasts reoriented towards the epidermis. (c) Overview of species mentioned in this review, together with their aquatic leaf morphology, biochemistry and established regulators. Superscript numbers in column 1 of (c) refer to the following: 1, van Veen *et al.* (2013); 2, Hsu *et al.* (2001); 3, Iida *et al.* (2016); 4, Kuwabara *et al.* (2003); 5, Momokawa *et al.* (2011); 6, Kim *et al.* (2018); 7, Nakayama *et al.* (2014); 8, Horiguchi *et al.* (2019); 9, Li *et al.* (2017); 10, Casati *et al.* (2000); 11, Rao *et al.* (2002); 12, Ueno (1998). ABA, abscisic acid; GA, gibberellin.

When confronted with a sudden change in environment, existing leaves have limited capacity to undergo drastic morphological changes. Therefore, the initiation of the development of either a terrestrial or an aquatic leaf is established early on in development at the shoot apex. Here we consider three main developmental changes from the terrestrial to aquatic transition perspective (Fig. 1), either the formation of narrow leaves or the formation of dissected leaves. Both leaf forms typically lack stomatal development.

### Elongated narrow aquatic leaves

When submerged, many amphibious species form new leaves that are longer and narrower. Sometimes the leaves are also pointed at the proximal end, forming an oblanceolate shape. Additionally, these leaves have a higher SLA, are thinner, lack stomata and have minimal cuticle development (Nakayama *et al.*, 2017). The advantage of producing a thin leaf without a cuticle is the reduction of the distance for inward diffusion of O_2_ and CO_2_ required for respiration and underwater photosynthesis. The exact importance of the aquatic leaf shape remains unclear, but a narrower leaf would have a thinner diffusive boundary layer (Box 1), further enhancing gas exchange with the environment. Detailed investigations in *Rumex palustris* estimated a 38‐fold reduction in CO_2_ diffusion resistance in aquatic leaves (compared with unacclimated terrestrial leaves) associated with higher photosynthesis rates and reduced photorespiration (Mommer *et al.*, 2005, 2006).

Amongst amphibious plants, abscisic acid (ABA) has emerged as a major regulator of leaf morphological alterations (Nakayama *et al.*, 2017). In *Marsilea quadrifolia*, the elongated aquatic leaf form requires low ABA conditions, and terrestrial leaves were created when submerged in water containing ABA. Subsequently, specific transcriptional ABA responses could already be observed at the shoot apex (Hsu *et al.*, 2001). The correlation between elevated ABA concentrations and the terrestrial leaf form was also found in *Potamogeton wrightii*. Here, even salinity stress‐induced ABA triggered terrestrial leaf formation underwater (Iida *et al.*, 2016). Such ABA‐dependent heterophyllous changes were also observed in *Ludwigia arcuata* where ABA concentrations were downregulated by ethylene accumulating in submerged tissues, as frequently observed in wetland species (Kuwabara *et al.*, 2003; Benschop *et al.*, 2005) .

The amphibious *Ranunculus trichophyllus* produces extremely thin, rounded aquatic leaves with enhanced abaxial and retarded adaxial development, in contrast to the thick, wide terrestrial leaf form (Kim *et al.*, 2018). Interestingly, its terrestrial relative, *Ranunculus sceleratus*, does not display such heterophylly. A transcriptome analyses of *R. trichophyllus* aquatic and terrestrial leaves revealed strong repression of genes associated with wax biosynthesis required for cuticle formation, and secondary cell wall and vascular development. Heterophyllic leaf development was determined by hormonal regulation of gene families involved in leaf polarity control, namely *HD‐ZIPIIIs* and *KANADI*. Submergence‐induced ethylene accumulation stimulated *KANADI*s required for abaxial development (Kerstetter *et al.*, 2001), whilst *HD‐ZIPIII*‐mediated adaxial development (McConnell *et al.*, 2001) was retarded via a submergence‐induced loss of ABA stimulation. *Ranunculus sceleratus* lacked these hormonal and transcriptional responses, suggesting that the changes in ABA/ethylene signalling and leaf polarity control are key evolutionary steps for aquatic adaptation (Kim *et al.*, 2018).

Leaf morphology is also strongly regulated by light quality cues (Momokawa *et al.*, 2011). The red : far‐red ratio (R : FR) rises with increasing water depth. Accordingly, low R : FR triggered terrestrial leaf formation in submerged *Rotolla hipuris,* with the converse being true for aquatic leaves upon emersion. Interestingly, R : FR values indicative of proximity to the water surface or aerial conditions required high blue light to facilitate underwater terrestrial leaf formation. At high R : FR values typical of deep flooding, blue light had no effect. Thus the integration of light quantity and quality can be critical in detecting water‐level fluctuations (Momokawa *et al.*, 2011).

### Dissected aquatic leaves

An extreme form of heterophylly is the formation of highly dissected leaves underwater, with reduced stomatal density and cuticle thickness. A narrow and dissected leaf might also facilitate better water flow around and through it, and so prevent mechanical stress. An increase in dissection index is found in many species across a wide range of phylogenetic lineages. The underlying mechanisms have recently been extensively investigated for species such as *Hygrophila difformis* and *Rorippa aquatica* (Nakayama *et al.*, 2014; Li *et al.*, 2017; Horiguchi *et al.*, 2019). While enhanced dissections are triggered by many cues (e.g. temperature and humidity), it can also be the default leaf shape. Common among all types of compound leaves and dissections identified thus far is the fact that leaves originate as simple primordia from the shoot meristem. In simple leaves, the primordia enter a differentiated state via a reduction in class I *KNOX* gene expression and an increase in class II *KNOX* gene expression. However, compound and dissected leaves can re‐enter an undifferentiated state by transient reactivation of class I *KNOX* genes (Bharathan *et al.*, 2002). This undifferentiated state then allows leaflet initiation, instigated by PIN1‐mediated auxin maxima (Barkoulas *et al.*, 2008). The separation of these leaflets requires *CUP SHAPED COTYLEDON* (*CUC3*)‐mediated suppression of growth between the auxin maxima, a process that is conserved across eudicots (Blein *et al.*, 2008). Among the Brassicaceae, leaf dissection is further determined by the presence of *REDUCED COMPLEXITY* (RCO) which locally suppresses growth at the sides of leaves to enhance dissection (Sicard *et al.*, 2014; Vlad *et al.*, 2014).

In *R. aquatica*, the submergence‐induced formation of the dissected leaf coincides with a decrease of class I *KNOX* and *CUC3* expression, analogous to the existing knowledge of compound leaf formation. Moreover, a drop in gibberellin (GA) concentrations was observed, and *KNOXI* genes are known to suppress GA biosynthesis. Subsequently, exogenous GA application or biosynthesis inhibition led to the reversal or exaggeration of leaf dissection, respectively (Nakayama *et al.*, 2014). However, hormonal investigation of submergence‐induced leaf dissection in *H. difformis* found contrasting effects of GA compared with *R. aquatica* (Li *et al.*, 2017). Here leaf dissection was predominantly driven by ethylene and low ABA concentrations. Although the molecular machinery of leaf dissection is considered conserved across species, two contrasting signalling behaviours were identified here.

### Development of stomatal density

The aquatic leaf has a strongly reduced stomatal density and cuticle thickness. Indeed, detailed molecular investigation in *R. trichophyllus* identified a strong downregulation of stomatal developmental genes underwater, some of which have been lost altogether in aquatic plants (Olsen *et al.*, 2016; Kim *et al.*, 2018). The underwater regulation of stomatal density and cuticle typically goes hand in hand with that of leaf shape, namely via ethylene, low ABA and/or high R : FR (Kuwabara *et al.*, 2003; Momokawa *et al.*, 2011; Iida *et al.*, 2016; Kim *et al.*, 2018) . A thick cuticle in terrestrial leaves, which have higher ABA concentrations than their aquatic counterparts, agrees with the role of ABA in mediating drought responses, which includes strengthening the cuticle (Cui *et al.*, 2016). However, the signals linked to stomatal development of amphibious heterophylly do not always align with patterns commonly observed. Stomatal density increases with high light and low CO_2_ availability, and is signalled through systemic leaves (Casson & Hetherington 2010). Although light intensities follow the same trend for heterophylly, the low CO_2_ availability underwater is not translated into high stomatal density. Likewise, low ABA concentrations and high R : FR are also known to increase stomatal density (Boccalandro *et al.*, 2009; Tanaka *et al.*, 2013; Jalakas *et al.*, 2018), whereas in amphibious heterophyllous plants, low ABA and high R : FR decrease stomatal density (Kuwabara *et al.*, 2003; Momokawa *et al.*, 2011; Iida *et al.*, 2016; Kim *et al.*, 2018). Thus, the aquatic developmental programme appears to override routine terrestrial regulatory networks determining leaf formation and stomatal density.

## III. Underwater photosynthesis

**Figure 2:**
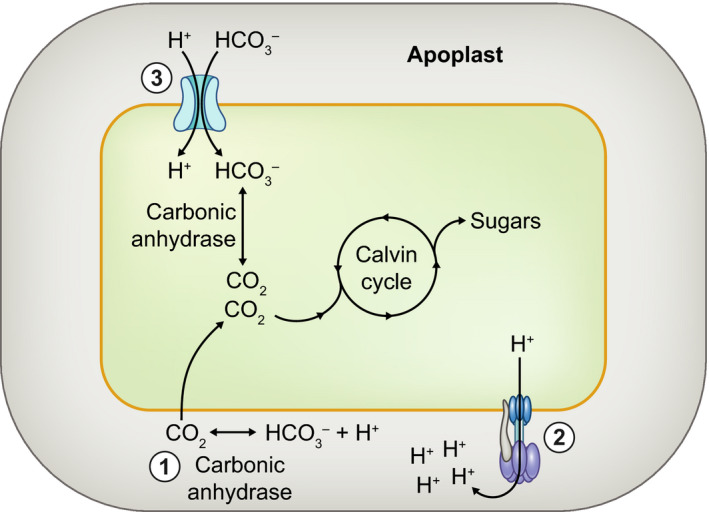
Bicarbonate utilization routes in angiosperms. In water, the predominant form of inorganic carbon is the membrane‐impermeable bicarbonate (HCO_3_^–^) ion. Successful underwater photosynthesis therefore requires bicarbonate uptake mechanisms, broadly categorized into three routes, depicted in the schematic: (1) an apoplastic carbonic anhydrase converting HCO_3_^–^ to CO_2_; (2) apoplastic acidification by H^+^‐ATPases, which shifts the chemical equilibrium towards CO_2_; and (3) symporters facilitating HCO_3_^–^ import into the cytosol. CO_2_ produced via these routes can ultimately be fixed via the Calvin cycle, eventually resulting in carbohydrate biosynthesis.

In air, gaseous CO_2_ diffuses relatively easily through the leaf. But when submerged, plants need to access the dissolved inorganic carbon (DIC). Between pH 7 and 10, CO_2_ availability is limited and HCO_3_^–^ is the dominant DIC form (Pedersen *et al.*, 2013). This poses an additional problem for submerged plants, as HCO_3_^–^, unlike CO_2_, does not easily cross lipid membranes. It is not surprising, therefore, that many aquatic plants have HCO_3_^–^ uptake mechanisms (Maberly & Madsen, 2002; Yin *et al.*, 2017). Various HCO_3_^–^ uptake strategies and CCMs have been characterized in cyanobacteria, algae, seagrasses and other higher plants (Poschenrieder *et al.*, 2018). In angiosperms, three forms can generally be distinguished (Fig. 2). First, the conversion of HCO_3_^–^ to CO_2_ by apoplastic carbonic anhydrases (CAs). Second, an H^+^‐ATPase‐mediated acidification of the apoplast and diffusive boundary layer, which pushes the CO_2_/HCO_3_^–^ equilibrium towards CO_2_. Third, a symporter‐mediated cotransport of HCO_3_^–^/H^+^, and subsequent HCO_3_^–^ dehydration to CO_2_ via cytosolic CAs. A variety of metabolic routes have been identified in aquatic plants to subsequently fix the acquired HCO_3_^–^/CO_2_. For example, the true aquatics (Box 1) *Hydrilla verticillata* and *Egeria densa* can switch between C_3_ and C_4_ photosynthesis underwater and can do so even in a single cell (Casati *et al.*, 2000; Rao *et al.*, 2002).

Few studies report photosynthetic adaptation to submergence in amphibious plants. The aquatic leaves of *R. palustris* clearly had better photosynthetic capacity underwater than did terrestrial leaves (Mommer *et al.*, 2005). In the heterophyllous amphibian *H. difformis,* a combination of biochemical and anatomical leaf adaptations facilitates underwater photosynthesis (Horiguchi *et al.*, 2019). Submergence triggered the formation of highly dissected aquatic leaves with substantial O_2_ production underwater. By contrast, submerged terrestrial leaves struggled to capture inorganic carbon, regardless of illumination. The decreased photosynthesis underwater and subsequent excess energy were linked to high oxidative stress in these leaves. Aquatic leaves had a high capacity to utilize HCO_3_^–^, which was absent in their terrestrial counterparts. Specific inhibitors were used to discern the mechanism for HCO_3_^–^ uptake in aquatic leaves. Interestingly, neither the inhibition of the apoplastic CA nor the HCO_3_^–^/H^+^ symport affected underwater photosynthesis. Significant photosynthesis impairment was observed only when intracellular CA activity was blocked. These observations imply that submerged leaves of *H. difformis* can import HCO_3_^–^ into the cell without H^+^ cotransport (Horiguchi *et al.*, 2019). Although common amongst true aquatic species, the extent of HCO_3_^–^ utilization amongst amphibious plants is currently unknown.

In true aquatics, CCMs such as the C_4_ system, can be induced by a several factors, such as photoperiod, low CO_2_ availability and ABA (Casati *et al.*, 2000; Rao *et al.*, 2002). The amphibious sedge *Eleocharis vivipara* exhibits an aquatic Kranz‐less C_3_ form and terrestrial C_4_‐like traits with Kranz anatomy (Box 1). The terrestrial form can be imposed on the aquatic leaf by ABA application and is considered as a stress signal (Ueno 1998) . Interestingly, in *H. difformis* biochemical HCO_3_^–^ usage, could be mimicked by application of ethylene or prevented by blocking ethylene perception. Even existing terrestrial leaves were sensitive to ethylene and submergence and achieved an intermediary capacity of HCO_3_^–^ usage (Horiguchi *et al.*, 2019). The importance of flooding‐specific cues, such as ethylene, is also apparent from work on *R. palustris*, where the morphological adaptations to submergence can also be induced by low light conditions. However, these do not yield any photosynthetic benefit, as does a true aquatic leaf (Mommer *et al.*, 2005).

## IV. Conclusions and future challenges

Amphibious plants are truly shape shifters, adjusting their morphology and physiology to adapt to fluctuating environments. They have provided crucial insights into developmental regulatory networks underlying leaf plasticity. However, while some consistent regulatory factors (e.g. ABA and ethylene) are recognized, there have also been contradictions (e.g. GA regulation of leaf dissection), and much remains to be discovered regarding other cues such as light, temperature and abaxial dominance in narrow leaves. This will require a greater use of amphibious species for exploring the molecular regulation of adaptive plasticity to water extremes. Given the increased fluctuations in water stress associated with climate change, understanding such adaptations will be important if we are to engineer resilient crops (Voesenek *et al.*, 2014). The plant’s current capacity to enhance underwater photosynthesis in existing terrestrial leaves of *H. difformis* is a good sign that such traits might, at some point, be transferable to crop species.
